# Effects of dexamethasone on the EC50 of remifentanil combined with dexmedetomidine achieving analgesia during pancreatic extracorporeal shockwave lithotripsy: a prospective, randomized and controlled study

**DOI:** 10.1186/s12871-024-02742-z

**Published:** 2024-10-10

**Authors:** Yu Guo, Jun Lu, Bo Li, Chang-Li Wang, Jia-Feng Wang, Xiao-Ming Deng

**Affiliations:** Faculty of Anesthesiology, Changhai Hospital, Naval Medical University, 168 Changhai Road, Shanghai, 200433 China

**Keywords:** Dexamethasone, Pancreatic extracorporeal shock-wave lithotripsy, Remifentanil, Pancreatic stones

## Abstract

**Background:**

In addition to their classic genomic effects, glucocorticoids also manifest rapid non genomic effects. We speculate that dexamethasone has the potential prompt onset of analgesic effects. The objective of this study is to investigate the influence of a single preoperative dose of dexamethasone on the half maximal effective concentration (EC50) of remifentanil when combined with dexmedetomidine for pain relief during pancreatic extracorporeal shockwave lithotripsy (P-ESWL).

**Methods:**

A total of 60 patients undergoing P-ESWL were enrolled and randomized at 1:1 ratio into the dexamethasone (DXM) group and the placebo group. Before anesthesia induction, patients in DXM group received an intravenous injection of 8 mg dexamethasone, while subjects in placebo group received an equal dose of physiological saline. Monitored anesthesia care (MAC) was performed based on remifentanil in combination with dexmedetomidine. Remifentanil was administered by TCI with an initial target concentration of 2.5 µg/mL for both groups. A positive response was defined as that VAS score > 3 by the patient at any time during the procedure. Subsequent target concentrations were adjusted by Dixon up-down sequential method, where dose modifications were performed by 0.3 ng/mL intervals, based on the response of the previous patient. The EC50 of remifentanil for pain relief during P-ESWL treatment was calculated using Dixon’s up-and-down method. Hemodynamic variables, oxygen saturation and adverse events were also recorded.

**Results:**

Dixon up-and-down method revealed that the EC50 of remifentanil was significantly higher in placebo group (2.65 ± 0.28 ng/mL) than in DXM group (2.02 ± 0.23 ng/ml) (*P *< 0.001). Hemodynamic parameter exhibited a significant decrease in mean arterial pressure (MAP) and heart rate (HR) before and after induction in placebo group; however, data of the two groups were comparable (*P*>0.05). Less adverse events occurred in DXM group, including the incidence of postoperative nausea and vomiting (PONV) and analgesia requirement with in the first 24 h following the procedure at ward.

**Conclusion:**

Dexamethasone exerted analgesic effects with a rapid onset, and patients received dexamethasone 8 mg preoperative had a lower required EC50 of remifentanil during P-ESWL. It is also associated with reduced PONV in addition to reduced postoperative analgesic consumption in the first postoperative 24 h.

**Trial registration:**

Registered in the Chinese Clinical Trial Registry (ChiCTR2300078171) on 30/11/2023.

## Background

Pancreatic extracorporeal shock wave lithotripsy (P-ESWL) is a safe and effective preferred therapy for pancreatic duct stones in patients with chronic pancreatitis [1]. In the anesthesia management of ESWL, the analgesia requirement is very strict to ensure immobility and comfort, optimizing shock wave delivery and improving targeting of stones during this painful procedure [2, 3]. Remifentanil is an effective short-acting µ receptor agonist, which has rapid metabolism and is easy to titrate and adjust its optimal dose without worrying about delayed recovery. Analgesia-based sedation with remifentanil alone has been utilized in anesthesia for urinary ESWL [2, 4]. However, the pain induced by ESWL for pancreatic stones seemed to be more severe than that experienced during ESWL for urinary stones and required greater analgesia [5]. High-dose opioids can effectively control pain but may induce chest wall rigidity and cause life-threatening respiratory depression. Simultaneously, the adverse reactions associated with opioid agonists, including nausea and vomiting, itching, and hyperalgesia, led to significant difficulties for patients [6, 7]. Therefore, these limitations have spurred the search for improved analgesic options.

Deep sedation during ESWL may lead to counter-productive due to deep respiratory excursions and sudden movements by patients. Dexmedetomidine is increasingly being used as a sedative for monitored anaesthesia care (MAC) because of its analgesic properties, “cooperative sedation”, and lack of respiratory depression [8, 9]. Previous studies have reported that MAC based on a combination of remifentanil and dexmedetomidine may be an ideal choice for outpatient surgical and painful treatments, such as hysteroscopy and Liposuction [10, 11]. However, there is currently no research investigated the effective concentration of remifentanil for pain relief during P-ESWL procedure on the basis of sedation with dexmedetomidine.

Glucocorticoids exert analgesic and anti-inflammatory effects via both non-genomic action within seconds to minutes and slow genomic pathways. The genomic pathway is responsible for a long-lasting effect on chronic pain, while non-genomic effect quickly reduces the release of glutamate and increases the levels of endocannabinoids and γ-aminobutyric acid, resulting in a significant reduction in neuronal excitability and anti-hyperalgesia [12]. Dexamethasone is a synthetic glucocorticoid, which is widely used in anesthesia practice because of its well-known analgesic and antiemetic properties in perioperative period [13, 14]. Previous studies have investigated the potential effect of a single intravenous injection of dexamethasone on postoperative analgesia and adverse events, but research on the rapid onset and analgesic effects of glucocorticoids during surgery is limited. In this study, we conducted a prospective, randomized, and controlled trial to determine the effect of dexamethasone on the quantitative-effect relationship of remifentanil in abolishing pain during P-ESWL procedures under MAC based on a combination of remifentanil and dexmedetomidine to provide a reference for clinical application.

## Methods

### Ethic

This prospective, randomized controlled clinical trial was approved by the Research Ethics Committee of Changhai Hospital of Naval Medical University with the approval number CHEC2023-116 (24/11/2023), and registered on the Chinese Clinical Trial Registry (ChiCTR2300078171) on 30/11/2023. The participants received a written and oral explanation about the protocol. Written informed consent was obtained from each participant or appropriate surrogate prior to enrollment. The trial was designed in accordance with the Consolidated Standards of Reporting Trials (CONSORT) reporting checklist.

### Study population

Sixty patients with American Society of Anesthesiologists physical status I or II, a body mass index 18 to 30 kg/m^2^, aged 18 to 60 years, and scheduled for primary P-ESWL treatment between November 2023 and January 2024 were enrolled. The exclusion criteria were as follows: known or suspected difficult airway (Mallampati score of 3 or 4 and a mouth opening < 3.5 cm), severe respiratory disease or obstructive sleep apnea, patients with poor blood sugar control, long-term use of analgesic drugs, allergy to study drugs.

Patients were eliminated during the study due to life-threatening adverse events induced by the P-ESWL procedure or incompleteness of their data. The inclusion criteria for P-ESWL indication were based on previous reports [15]. The right of each participant to refuse or withdraw from the study without giving reasons was respected.

### Randomization and blinding

Eligible individuals were recruited consecutively and randomly assigned to receive either dexamethasone or normal (0.9%) saline placebo at a 1:1 ratio using computer-generated randomisation with a block size of six. The randomization sequence were concealed by opaque sequentially numbered sealed envelopes. A investigator (Yu Guo) who did not participate in any anaesthetic care or data collection opened the sealed envelopes and prepared the drug in identical syringes according to the group allocation. The effect-site concentrations of remifentanil target-controlled infusions was also set by Yu Guo, according the previous patient’s response reported by the fixed attending anesthetist, who were blinded to group assignments. The fixed attending anesthesiologist administered the sedation procedure and another blinded research assistant collected the data. Patients in each group remained unaware of their intervention assignment.

### Trial protocol

The patients were educated to evaluate their pain using a 10-cm Visual Analogue Scale before surgery with 0 representing no pain and 10 representing the worst pain. Upon entering the operating room, standard monitoring was performed including electrocardiogram, noninvasive arterial pressure, oxygen saturation (SpO_2_), and respiratory rate (RR). Oxygen (4 L/min) was supplied through the nasal cavity, and the end-expiratory carbon dioxide tension and respiratory rate (RR) was monitored through one of the nostrils.

Patients in the DXM group received intravenous injection of 8 mg dexamethasone (4 ml, 2 mg/ml) before anesthesia induction, while those in placebo group received 4 ml normal saline. MAC was started and maintained by target-controlled infusion of remifentanil and a continuous infusion of dexmedetomidine. Remifentanil was administered using a Minto model effect-site target-controlled infusion pump (Orchestra Base Primea, Fresenius Vial, France). Dexmedetomidine infusion began with a loading dose of 0.5 ug/kg over 10 min, followed by a maintenance infusion of 0.5 mug/kg/h until the end of the procedure. All patients were asked to notify the anesthesia if they experienced pain (VAS>3) at any time during the procedure. Extracorporeal shock wave lithotripsy was initiated 3 min after reaching the set target for the effect-site concentration. Intraoperative sedation (or sleepiness) levels was assessed with the Ramsay Sedation Scale (RSS) ranging from 1 to 6 (1, agitated; 2, cooperative and oriented; 3, can respond to simple questions; 4, asleep, but with a quick reaction to stimulus; 5, asleep, arousable; 6, asleep, unarousable). During each P-ESWL therapeutic session, a maximum of 5000 shock waves were delivered at an intensity ranging from 1 to 6, with a frequency of 120 shocks per minute.

The initial effect-site concentration of remifentanil was set at 2.5 ng/ml, with increments or decrements of 0.3 ng/ml used as ideal concentration step according to Dixon’s up and down method. Inadequate analgesia was considered if there was a positive response (VAS > 3 or a complaint of insufferable pain by the patient), leading to an increase of 0.3ng/ml in the next target effect-site concentration of remifentanil. Conversely, if there was a negative response (VAS ≤ 3), the target concentration of remifentanil for the next patient would be decreased by 0.3 ng/ml. For patients who complained of unbearable pain during the P-ESWL, the target concentration of remifentanil was titrated to a specific concentration, which corresponds to a VAS score of less than 3, so as to offset unbearable pain. If the unbearable pain was not relieved after an increase of remifentanil, the patient will have the opportunity to receive anesthesia with intravenous propofol to achieve deep sedation or general anesthesia. In the event of respiratory depression (spontaneous respiratory rate less than 8 breaths per minute or SpO_2_ less than 90% lasting for 10 s), patients were awakened and encouraged to take deep breaths. Ventilation with a facial mask was provided if patients failed to wake up from sedation or maintain spontaneous breathing.

After completing P-ESWL, the dexmedetomidine and remifentanil infusions were discontinued, and patients were transferred to a post-anesthesia care unit (PACU) where they remained for at least 30 min. Additional analgesia was administered in the PACU and general ward if patients complained of moderate to severe postoperative pain. Patients were followed up for postoperative nausea and vomiting (PONV) and analgesia requirements for 24 h after P-ESWL in the ward.

### Outcome measures and data collection

Demographic data were extracted from the patient’s medical records. Sedation durations and.

total consumption of remifentanil werecollected at the end of surgery and the mean infusion rate was calculated by dividing the total dose by body weight and infusion time. The hemodynamic parameters (HR and MAP, RR) and RSS during sedation were recorded at intervals of 10 min until the end of the ESWL procedure. Adverse effects such as postoperative nausea and vomiting, pruritus, and additional analgesia if any were noted.

The EC50 of remifentanil for pain relief during P-ESWL in each group was the primary study outcomes. Secondary outcomes included vital signs, sedation levels, incidence of adverse effects, and the need for additional analgesia within the first 24 h after the procedure.

### Statistical analysis

According to a previous study, elucidation of EC50 by Dixon up-and-down method needed at least 6 crossover pairs, and simulation study conducted by Stylianou and Flournoy suggested the sample size of 20–40 patients can provide a stable estimation of the target dose in most situations [2, 16]. For this study, a simple size of 30 patients in each group were deemed sufficient to estimate the EC based on the up-down allocation method.

Statistical analysis was performed using SPSS 27.0 software. Continuous variables were analyzed using independent T-tests or Mann-Whitney U tests and presented as mean with standard deviation or median with range. Categorical variables were presented as numbers with percentages and compared using the chi-squared test or Fisher’s exact test. The EC50 of remifentanil was obtained by the mean value of the mid-point for each positive-to-negative pair. The Student’s t-test was used to compare EC50s. Additionally, probit method were also applied for the evaluation of EC50 of remifentanil. *P* values were two-sided and values less than 0.05 was considered significant.

## Results

**Fig. 1:**
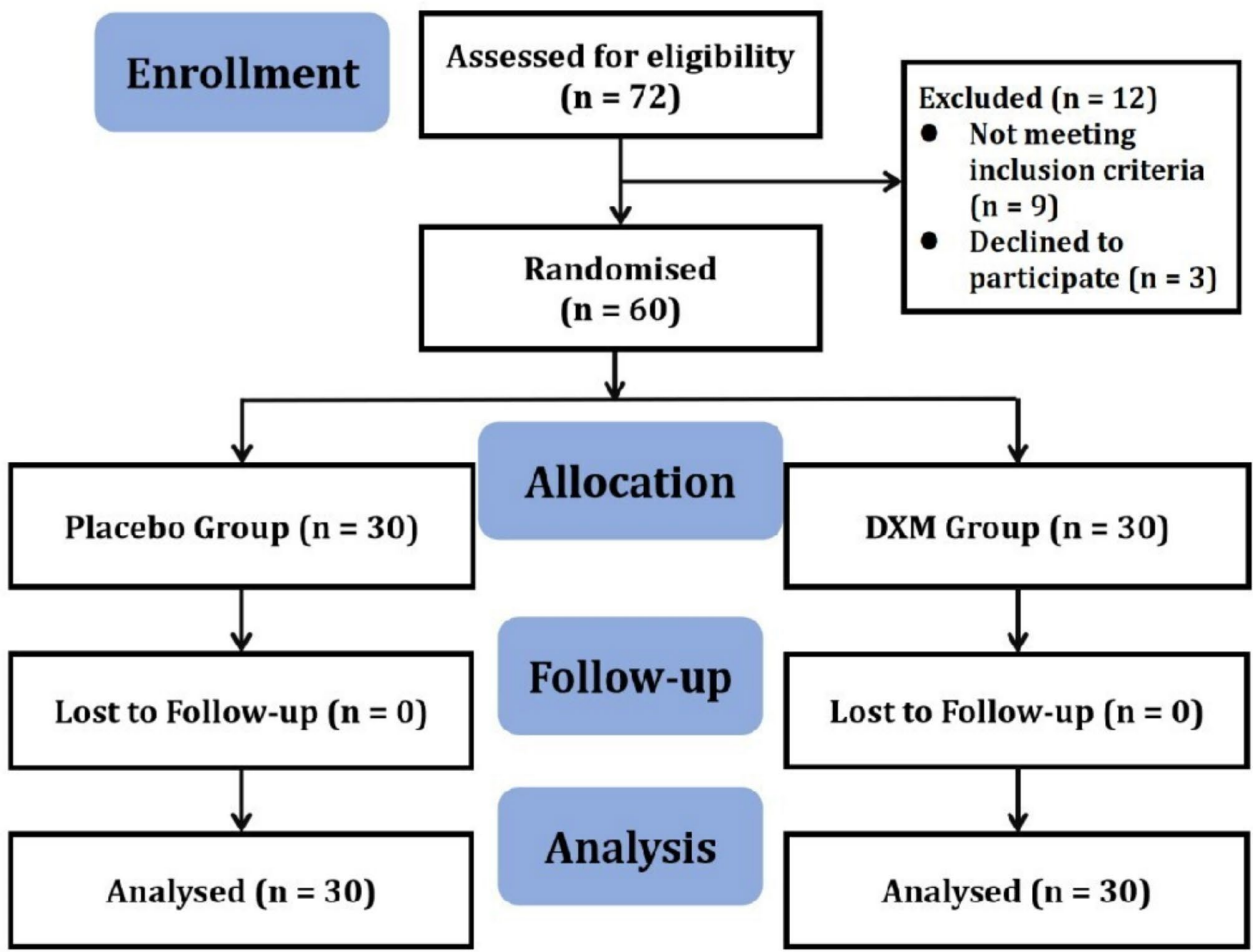
The consort flow chart

A total of 72 patients were screened, and 30 participants were randomly assigned to each group. All 60 participants completed the study without any dropouts (Fig. 1). The patient’s baseline characteristics were comparable between the two groups, with no statistically significant differences in age, gender, weight, height, BMI, diabetes and hypertension. The sedation duration was similar between the two groups (Table 1).

Demographic characteristics, health status and surgical details are displayed in Table 1.

**Table 1:** Baseline demographic and clinical characteristics of the patients

	Placebo group (*n* = 30)	DXM group (*n* = 30)	*p* value
Age (years)	44.1 ± 12.3	44.8 ± 9.83	0.79
Gender (female)	12 (40%)	12 (40%)	1
Height (cm)	168 ± 7.5	167.9 ± 8.1	0.95
Weight (kg)	60.8 (55.0–69.3)	61.7 (56.0–67.0)	0.64
BMI (kg/m2)	22.3 ± 2.4	21.6 ± 1.8	0.21
Diabetes	8 (26.7%)	6 (20%)	0.76
Hypertension	3 (10%)	2 (6.7%)	> 0.99
Duration of sedation (minute)	47 (46–48)	47 (46–48.25)	0.57
Total dose of remifentanil (µg)	342.2 (303.6–395.9)	276.0 (256.3–296.6)	< 0.001
Infusion rate of remifentanil (µg/kg/min)	0.118 ± 0.023	0.098 ± 0.019	< 0.001

Data are presented as the mean+-standard deviation (SD), median and interquartile range (IQR), or number (%) as appropriate

Abbreviation: BMI, body mass index; ASA, American Society of Anesthesiologists

### Primary outcome

**Fig. 2:**
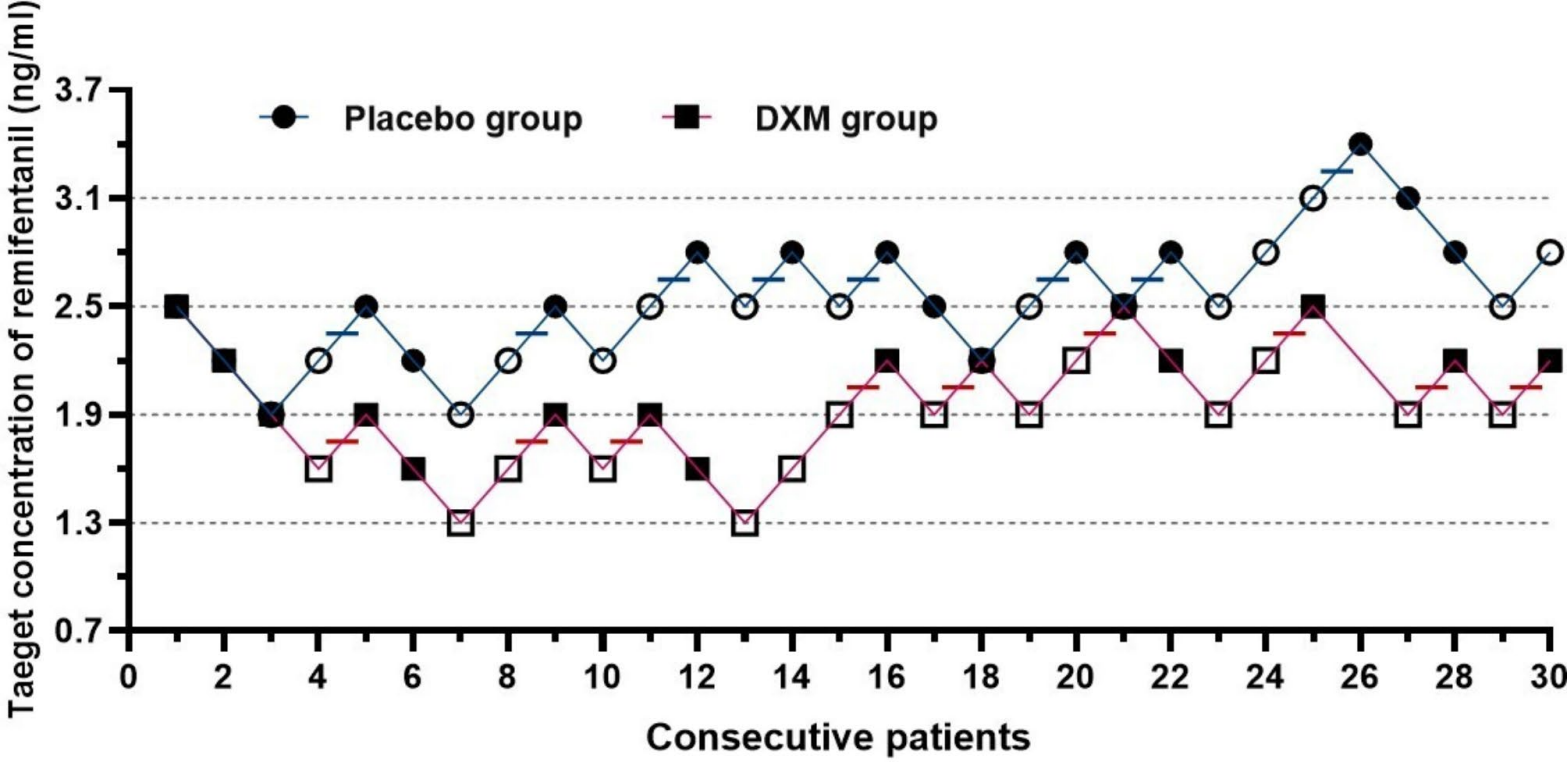
Effect-site concentration of remifentanil by Dixon’s method in placebo group and in DXM group. A negative response (VAS < 3) is denoted by a black solid circles or block; and a positive response is denoted by an open circle or block; Horizontal bars represent crossover midpoints (positive to negative)

Dose–response data for each patient obtained by Dixon’s up-and-down methods was displayed in Fig. 2. The figure illustrates the change in remifentanil target concentration between the initial patient and subsequent patients, with a positive response leading to increase in the remifentanil target concentration in next patient and vice versa. The EC_50_ of remifentanil estimated by Dixon’s up-and-down method was significantly lower in the DXM group (2.02 ± 0.23 ng/ml) than the placebo group (2.65 ± 0.28 ng/mL) (*P* < 0.001). The probit analysis showed that EC50 of remifentanil were 1.91 (95% CI 1.68–2.14) ng/ml in DXM group and 2.59 (95% CI 2.28–2.89) ng/ml in placebo group, respectively. Additionally, the total dose of remifentanil administered was recorded. The total dose of remifentanil in the DXM group was significantly lower than that in the placebo group (*P* < 0.001; Table 1). Furthermore, the mean infusion rate of remifentanil in the DXM group was significantly lower than that in the placebo group (0.098 ± 0.019 vs. 0.118 ± 0.023 µg/kg/min) (*P* < 0.001; Table 1).

### Hemodynamic parameters and RSS scores

**Fig. 3:**
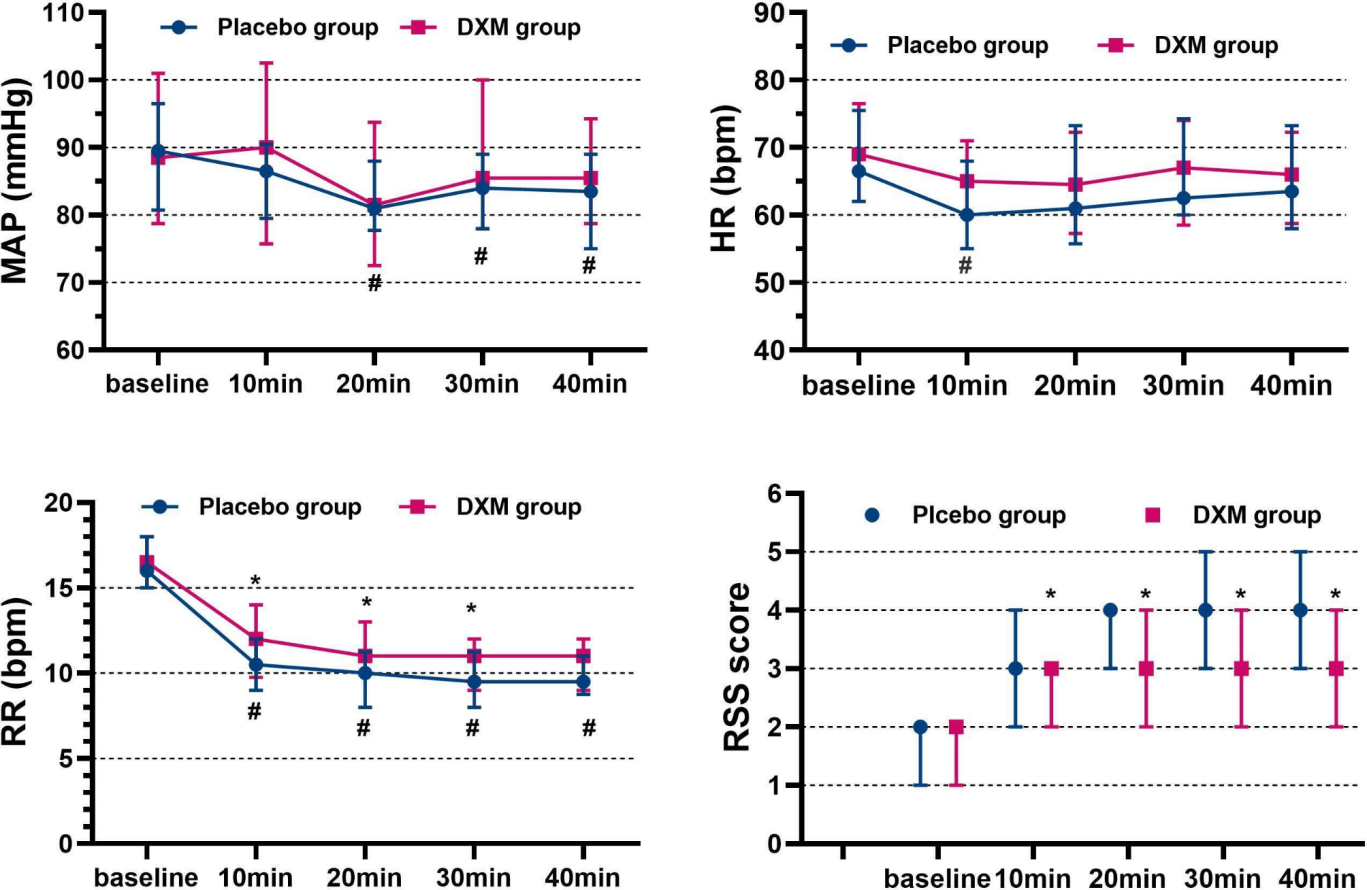
Hemodynamic parameters and RSS scores at five measurement points. MAP, HR, RR and RSS scores were presented as median (IQR) and absolute range. * *P* < 0.05, compared with placebo group, #*P* < 0.05, compared to baseline. Abbreviations: HR, heart rate; MAP, mean arterial pressure; RR, respiratory rate, RSS, Ramsay Sedation Scale

Baseline vital signs (HR, MAP, RR) and the sedation scores were comparable in both groups (Fig. 3). The comparison of MAP and HR values of the patients in the groups at the same timepoint showed no statistical differences (*P* > 0.05). The MAP at the 10th, 20th, 30th, and 40th min and HR at the 10th min in placebo group decreased significantly compared to those at baseline (*P* < 0.05). The MAP and HR in DXM group was relatively stable. The induction of sedation produced a significant decrease in RR values in both groups (*P* < 0.05). Also, the RR values of the placebo group were lower than that of the DXM group at 10th, 20th and 30th min (*P* < 0.05). RSS scores at almost all timepoint in both groups were significantly higher than the baseline values (*P* < 0.05). However, RSS scores were statistically lower in the DXM group than placebo group during the procedure (*P* < 0.05).

No episodes of bradycardia or hypotension were observed in either group, and only one patient in the DXM group experienced transient hypertension requiring intervention.

No patients required ventilatory support, and spontaneous breathing was maintained. Only 3 patients in the placebo group experienced respiratory depression (oxygen saturation < 90% for more than 10 s), which was addressed by instructing them to take a deep breath.

### Adverse events

No statistically significant differences were observed between the number of patients suffering from pruritus and chest wall rigidity in both groups [Table 2]. PONV incidence was quite comparable between the groups in PACU (*P*>0.05). However, the incidence of PONV was significantly higher in the placebo group (40%) compared with the DXM group (10%) with in the first 24 h after arrival to the ward (*P*<0.05). Three patients in placebo group required analgesia and one in DXM group needed analgesia in the PACU (*P*>0.05). At 24 h after surgery in ward, the rate of patients who required analgesia was 20% in the placebo group and none in the DXM group ( *P*<0.05).

**Table 2:** Adverse events in the perioperative period

	Placebo group (*n* = 30)	DXM group (*n* = 30)	*p* value
Chest wall rigidity	0	0	-
Pruritus	5 (16.7)	1 (3.3)	0.19
PONV in PACU	6 (20)	1 (3.3)	0.10
Analgesia requirement in PACU	3 (3.3)	0	0.24
PONV in ward	12 (40)	3 (10)	0.02
Analgesia requirement in ward	6 (20)	0	0.02

Values are expressed as the n (%). PONV: post-operative nausea and vomiting. PACU: post-anesthesia care unit

## Discussion

In this study, we evaluated the EC50 of remifentanil combined with dexmedetomidine for pain management during P-ESWL with or without dexamethasone (8 mg) before anesthesia. The co-administration of dexamethasone significantly reduced remifentanil EC50 by 23.7% (2.02 ± 0.23 vs. 2.65 ± 0.28 ng/mL), compared to the placebo group. In additional, the administration of dexamethasone was associated with reduced intraoperative remifentanil consumption and infusion rate, as well as decreased postoperative analgesic requirements and complications such as PONV in patients undergoing P-ESWL.

The type of sedation was based on the preference and experience of anesthesiologists in the different centers. We used propofol combined with remifentanil for sedation in P-ESWL, demonstrating better amnesia and comfort, but higher risk of respiratory depression or even apnea and hemodynamic depression. Also, there are more unwanted involuntary movements and poor compliance of breath holding during procedure, because of excessive sedation and not sufficient analgesic agent. The preferred type of intravenous sedation for P-ESWL procedure is light to moderate sedation (“conscious sedation”) for quality of care or safe practice. Remifentanil is a potent analgesic with rapid onset and offset, making it easily adjustable to the required level of pain relief. These characteristics make remifentanil suitable for use in TCI during monitored anesthesia care and have been successfully used for pain relief during ESWL [17]. To address pre- or intra-operative anxiety, intravenous infusion of dexmedetomidine was used to alleviate anxiety and provide sedation [18]. The hemodynamic effects of dexmedetomidine are influenced by the loading dose and infusion rate. In the present study, significant hypotension or bradycardia was not observed, which may be attributed to the low loading dose of dexmedetomidine.

The differences in remifentanil EC50 between the two groups demonstrate the rapid onset of analgesic effect of dexamethasone. Glucocorticoids have both slow genomic effects and rapid nongenomic effects. Previous studies have focused on the slow genomic effects of a single dose of dexamethasone before induction or during surgery, which have been shown to be effective in reducing postoperative pain, opioid consumption, and the need for rescue analgesia [19–21]. However, the rapid analgesic effect of dexamethasone during surgery has not been demonstrated before. The rapid nongenomic action of dexamethasone appears quickly and is thought to be achieved through interactions with components of cellular membranes, membrane steroid receptors, or cytosolic glucocorticoid receptors [22, 23].

Our results contradict previous studies that reported higher remifentanil infusion rates in patients undergoing total knee arthroplasty under general anesthesia who received dexamethasone shortly before surgery [24]. It is possible that the adjustment of remifentanil infusion rates is subjectively based on clinical assessment by the anesthetist, taking into account the patient’s vital signs such as blood pressure and heart rate. As the authors stated, the hemodynamic effects of dexamethasone could explain their findings.

The optima analgesic dose of dexamethasone in the perioperative period has not been determined. A dose of dexamethasone 8 mg IV rather than a dose of 4 mg may be better for PONV prophylaxis [25]. Preoperative dexamethasone 8 mg has been shown to be effective in improving the postdischarge quality of recovery in addition to reducing nausea, pain, and fatigue [26]. Hyperglycemia may be one of the annoying side effects of of glucocorticoids, particularly in diabetic patients. Murphy et al. demonstrated that blood glucose concentrations did not differ significantly between the groups receiving dexamethasone 8 mg and those receiving saline at any measurement time [27]. A randomized controlled study showed that dexamethasone 8 mg did not induce greater hyperglycemia, and maximal perioperative blood glucose concentrations in patients with diabetes were related to baseline HbA1c values in a concentration-dependent manner [28]. None of the patients who receiving 8 mg dexamethasone required additional insulin therapy, including the diabetic patients. This could be argued by the fact that the small number of diabetic patients included in this study. Further studies with more sample size and longer follow-up are indicated to assess the safety of dexamethasone applied in these patients.

Dexamethasone have been shown to reduce postoperative pain in many types of surgery. The assessment of pain indicated that preoperative intravenous glucocorticoids can reduce postoperative pain following P-ESWL. Edema and hemorrhage in the pancreas are mainly side effects of shock wave lithotripsy. Glucocorticoids reduce pain by inhibiting prostaglandin synthesis and reducing vascular permeability, resulting in decreased tissue edema. This can be attributed to the activation of various pain control pathways, in addition to the direct effect of dexamethasone on its receptors [29]. Furthermore, dexamethasone is a well-known antiemetic via an activation of the glucocorticoid receptors in the bilateral nuclei tractus solitary in the medulla [30, 31]. We confirmed this in our study, with an almost 30% reduction in the frequency of PONV compared to placebo.

The currently study suffers some limitations. Firstly, we did not investigate the side effects of dexamethasone, particularly in conscious sedation patients, such as pudendal pruritus and postoperative headache. Additionally, the sample sizes were small, which may have contributed to the lack of significant differences in secondary outcomes between the two groups. Finally, clinical heterogeneity, such as the etiology of pancreatitis, features of the stone, and location of the stone, can affect the outcomes.

## Conclusion

In conclusion, our study demonstrated that the EC50 of remifentanil needed for patients undergoing P-ESWL under MAC with remifentanil and dexmedetomidine was 2.02 ± 0.23 ng/ml and 2.65 ± 0.28 ng/mL in the presence or absence of dexamethasone, respectively. This indicates that dexamethasone has a rapid onset of analgesic effect. Furthermore, a single dose of dexamethasone 8 mg shortly before surgery can reduce remifentanil consumption during surgery and postoperative rescue analgesic requirements and the incidence of PONV.

## Data Availability

The datasets generated and analyzed during the present study are available from the corresponding author on reasonable request. Xiao-Ming Deng, E-mail: dengphd@smmu.edu.cn.

## Abbreviations

EC50: Half maximal effective concentration
P-ESWL: Pancreatic extracorporeal shockwave lithotripsy
MAC: Monitored anesthesia care
TCI: Target-controlled infusion
CI: Confidential interval
DXM: Dexamethasone
MAP: Mean arterial pressure
HR: Heart rate
PONV: Postoperative nausea and vomiting
CONSORT: The Consolidated Standards of Reporting Trials
ASA: American society of Anesthesiologists physical status
VAS: Visual analogue scale
BMI: Body mass index
